# Recent Advances in Stimuli-Responsive Microgels and Their Biomedical Applications

**DOI:** 10.3390/molecules30224457

**Published:** 2025-11-19

**Authors:** Hongtao Zhang, Yongfeng Gao

**Affiliations:** 1 School of Chemistry and Chemical Engineering, Qinghai Normal University, Xining 810008, China; 2 Department of Chemistry, University of Alberta, Edmonton, AB T6G 2G2, Canada

**Keywords:** stimuli-responsive microgels, functional monomer, cross-link chemistry, post-functionalization

## Abstract

Stimuli-responsive microgels, smart polymeric particles at a micro- to sub-micro scale that are capable of undergoing reversible changes in response to external triggers, have emerged as versatile tools in the field of biomedical research. The review begins by emphasizing the importance of precise control over microgel properties, such as size, composition, and responsiveness, to harness their full potential. It delves into various synthetic methodologies, including precipitation polymerization, emulsion polymerization, and microfluidic techniques. The versatility of microgels, combined with their ability to respond to specific stimuli, holds great promise for tailored biomedical applications. By dissecting the responsive attributes of microgels and unraveling the intricate structure-property relationships they embody, this review elucidates the invaluable contributions of these remarkable smart materials to diverse biomedical applications, paving the way for future advancements in the field.

## 1. Introduction

Microgels, comprising cross-linked polymeric particles primarily composed of water-soluble or swellable polymer chains, are essentially micro- to sub-micron scale counterparts of hydrogels [1,2,3]. These microgels possess exceptional attributes, such as a high water content, biocompatibility, and favorable mechanical characteristics. Particularly noteworthy is their rapid response to environmental variations, which surpasses the reactivity of macroscopic or bulk hydrogels. These features render microgels exceptionally effective for a diverse range of medical applications [4,5]. Thanks to their significantly expanded interfacial area, microgels exhibit enhanced exchange rates, endowing them with distinct advantages for polymer-based drug delivery systems [6,7,8,9]. Their adaptability extends further, offering tunable dimensions spanning from nanometers to micrometers. This expansive range provides a significant surface area, facilitating multivalent bioconjugation, and an internal network that seamlessly accommodates the incorporation of biomolecules. In light of the current and forthcoming applications of microgels, precise control over their properties is imperative to unlock their complete potential. These attributes encompass the need for stability, ensuring extended circulation within the bloodstream, the incorporation of novel functionalities to facilitate additional bioconjugation, the precise control over particle size with consistent diameters, and inherent biodegradability. This biodegradability is essential for achieving sustained drug release over a predefined duration while maintaining the convenience of removing empty devices when necessary.

In the context of drug delivery systems, the development of effective microgel-based devices hinges on meeting several specific requirements. The foremost criterion to consider is the biocompatibility and biodegradability of the microgels [4,10]. Particularly in the development of systems or materials intended for medical applications, the primary prerequisite is compatibility with the biological environment or the human body. This compatibility is substantiated by the non-toxic nature of these materials and their ability to be easily metabolized. Extensive research efforts have been directed towards the development of responsive microgels featuring functionalized degradable groups or cross-linkers to fulfill this essential criterion, such as peptides [11], anhydrides [12], polyglycerol [13], disulfides [14], acetals [15], poly(3-hydroxybutyrate) [16], and polyphosphoesters [17]. In external environments, hydrogel or microgel particles cross-linked using these linkages undergo degradation, ultimately transforming into water-soluble polymers. The second critical criterion centers around the microgels’ responsiveness to both external and internal stimuli, enabling reversible or non-reversible structural changes. One of the most common reversible phase transition behaviors involves changing their volume, transitioning from a polymeric solution (in the swollen state) to a rigid particle (in the collapsed state) [18,19,20]. This transformation is initiated by conformational changes occurring within the sub-chains that connect adjacent cross-linking points within the gel network. It results from the interplay between repulsive intermolecular forces that promote the expansion of the three-dimensional polymer network, comprising loosely cross-linked polymeric chains constituting the microgels, and attractive forces that induce contraction. Swelling occurs when ionic repulsion and osmotic forces overcome the attractive forces, which may include hydrogen bonds, van der Waals interactions, and hydrophobic forces.

Microgels commonly display sensitivity to various environmental stimuli, which includes physical factors like temperature, ionic strength, and the presence of magnetic or electric fields. Additionally, they can respond to a range of chemical stimuli, such as alterations in pH, the introduction of specific ions, or the presence of particular molecules [21,22,23,24]. Furthermore, microgels are also capable of responding to biotechnological stimuli, such as enzymatic substrates, affinity ligands, or cell receptors. This versatile responsiveness makes microgels a highly adaptable platform for a wide range of tailored application [25,26,27].

In this comprehensive review, we delve into the intricacies of designing stimuli-responsive microgels and explore their multifaceted applications within the realm of biomedical research. By dissecting the responsive attributes of microgels and unraveling the intricate structure-property relationships they embody, we aim to elucidate the invaluable contributions of these remarkable smart materials to diverse biomedical applications.

## 2. Design Strategies of Stimuli-Responsive Microgels

In contrast to natural polymers, synthetic polymers provide the advantage of precise control and tunability of composition, multi-level degradability, and the ability to create well-defined microstructures. These attributes have sparked growing interest in the development of innovative stimuli-responsive microgels for various biomedical applications [2,28,29,30]. Several important synthetic polymers with stimuli-responsive properties play a crucial role in the construction of microgel systems. Selecting the right synthetic strategy is essential for crafting microgels with precisely controlled dimensions, minimal polydispersity, and customized physical and chemical properties, all of which are key factors in ensuring their effective utilization for specific applications.

**Figure 1:**
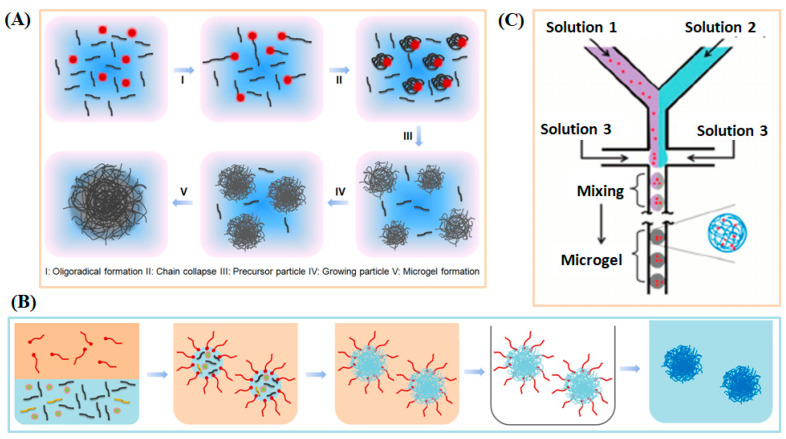
The synthesis approaches for generating stimuli-responsive microgels/nanogels from synthesis polymers. (**A**) Precipitation polymerization. (**B**) Emulsion polymerization. (**C**) Microfluidic method.

Synthetic methodologies for producing functional microgels can generally be categorized as: (a) precipitation polymerization [31,32,33]; (b) emulsion polymerization [34]; and (c) microfluidic method [35], and summarized in Figure 1. Precipitation polymerization is a widely used technique for producing monodisperse microgel particles with tunable sizes (as illustrated in Figure 1A). This method has been extensively utilized in the synthesis of responsive microgels, including those based on Poly(N-isopropylacrylamide) (PNIPAM) [36], Poly(*N*-vinylcaprolactam) (PVCL) [37], Poly(*N*,*N*-diethylacrylamide) (PDEAAm) [38], and other variants of PNIPAM-based microgels. PNIPAM has emerged as a prominent thermo-responsive polymer and has been extensively studied due to its remarkable properties. PNIPAM exhibits a lower critical solution temperature (LCST) at approximately 32 °C, which closely aligns with physiological temperatures. Beyond its LCST, PNIPAM chains undergo a distinctive transition, transitioning from an extended (solvated) random coil configuration to a compact (desolvated) globular conformation. This coil-to-globule transition for individual polymer chains can be finely tuned through thermodynamic adjustments in the polymer composition. This method is well suited for generating relatively monodisperse microgels because particle nucleation occurs within a narrow time window. Once nuclei form, further growth proceeds primarily by the diffusion of monomers and oligomers to existing particles rather than the creation of new ones. This controlled nucleation-growth mechanism limits secondary particle formation and contributes to a narrower size distribution compared to other systems. It accommodates both batch and continuous modes of production, ensuring precise control over microgel sizes and maintaining narrow particle size distributions. This method also facilitates the incorporation of various co-monomers and functional nanomaterials, empowering the design of diverse microgel architectures with tailored chemical compositions. However, it often produces broader particle-size distributions and provides less control over uniformity. It is important to acknowledge that the elevated temperatures involved in precipitation polymerization may limit its utility for incorporating temperature-sensitive biomolecules.

**Figure 2:**
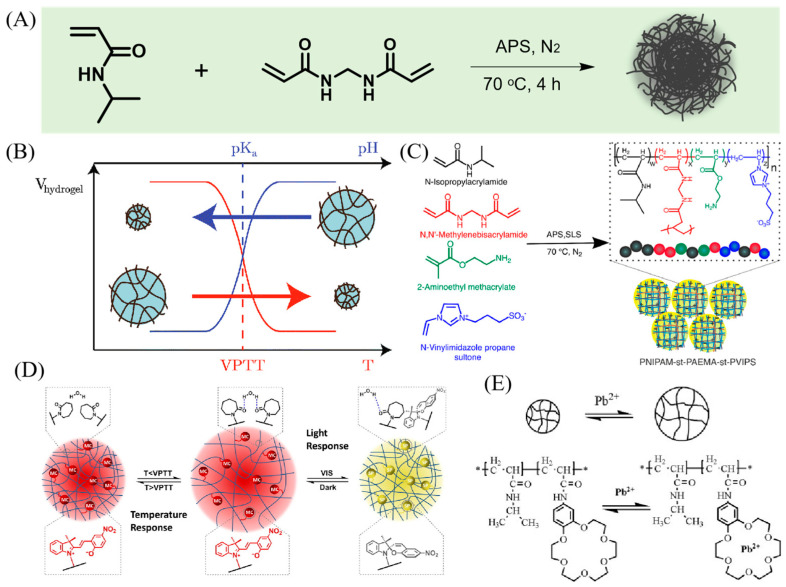
(**A**) Synthesis route of thermo-responsive PNIPAM microgel. (**B**) Diagram of volume change of temperature and pH-responsive microgel. Reprinted with permission from Ref. [54] (**C**) Schematic illustration of an example of zwitterionic microgel. Reprinted with permission from Ref. [55] (**D**) Light responsive microgel. Reprinted with permission from Ref. [56] (**E**) Metal ion responsive microgels system. Reprinted with permission from Ref. [57].

An alternative approach is the water-in-oil emulsion method (depicted in Figure 2B), which involves polymerizing monomers and co-monomers within water droplets. These droplets are stabilized by surfactants in the presence of a cross-linker. This versatile method is not confined to responsive polymers and is well-suited for crafting microgels and nanogels using both hydrophilic and hydrophobic polymer materials. Emulsion polymerization offers efficient production of small and relatively monodisperse particles, with good flexibility for incorporating functional monomers, but the reliance on surfactants and stabilizers can introduce impurities and necessitate additional purification for biomedical use.

Additionally, the microfluidic technique (illustrated in Figure 2C) has emerged as a powerful tool for generating a diverse array of microgels, ranging in size from micrometers. In this approach, monodisperse droplets of monomers or polymers are formed through the manipulation of liquid streams, followed by subsequent physical or chemical cross-linking processes. A notable advantage of this approach is its capability to generate microgel particles with larger dimensions, typically ranging from 1 to 30 µm. This precise control over size and monodispersity is achieved by manipulating the viscosities, polarities, and flow rates of the involved fluids. Furthermore, the integration of various co-monomers, biomolecules, or the encapsulation of nanoparticles or cells offers the flexibility to tailor the properties of these microgels, leading to the creation of intricate and multifunctional microgel architectures. In addition to its advantages, microfluidic fabrication also faces several limitations. Scaling up production remains challenging due to throughput constraints and device complexity. Furthermore, the use of oils and surfactants may introduce biocompatibility concerns, particularly for biomedical applications. Compared with microgels obtained by precipitation polymerization, microfluidic-generated microgels typically exhibit narrower size distributions and more uniform structures but may differ in surface chemistry and residual additives. These distinctions and limitations should be considered when selecting an appropriate fabrication method.

In the following section, we will explore the methodologies employed to engineer smart microgels endowed with functional properties. This intricate process entails a fusion of functional monomers, cross-linkers, and post-functionalization techniques, leading to the creation of intelligent microgels.

### 2.1. Functional Monomer

The responsiveness of microgels to external stimuli is intricately linked to their distinctive chemical structures, which dictate their ability to react to changes in environmental conditions. These responsive properties emerge from the precise chemical compositions inherent to microgels. Among the earliest and most widely recognized stimuli-responsive microgels is the thermo-responsive NIPAM microgel [32,39]. Besides NIPAM, N-isopropylmethacrylamide (NIPMAM) [40,41,42] and N-vinylcaprolactam (VCL) [43,44] can also be used as monomer for thermoresponsive microgels. Furthermore, the integration of various functional monomers into stimuli-responsive microgels allows them to respond to a diverse range of stimuli. Charged comonomers (cationic [45,46] and anionic [47,48,49]) can endow microgels with sensitivity to pH. Microgels can contain either a single type of charge (either cationic or anionic) or both types of charges simultaneously (zwitterionic [50,51]). Light responsive monomers (for example: 4-[(4-methacryloyloxy) phenylazo] benzenesulfonic acid [52] and nitrobenzyl methacrylate [53]) can introduce the light-responsive function into microgels. Some of the monomers can be responsive to specific heavy metal ions, Pb^2+^ for an example [54]. These monomers can impart specific metal ion-responsive properties to microgels.

To impart thermosensitive properties to microgels, they are commonly synthesized using monomers like NIPAM, NIPMAM, and VCL. The earliest thermoresponsive microgel is reported by R.H. Pelton and P. Chibanate [32], as illustrated in Figure 2A. A systematic exploration was carried out by altering the ratio of NIPAM monomers to cross-linkers during microgel synthesis. This investigation aimed to assess their thermoresponsive characteristics at varying compositions of NIPAM monomers. Microgels constructed using NIPAM exhibit a distinct thermoresponsive profile. This behavior can be attributed to the presence of both hydrophobic and hydrophilic elements within the NIPAM monomer. The hydrophilic segments engage in hydrogen bonding with the surrounding solvent water, resulting in swelling below the LCST. Conversely, the hydrophobic portions interact via hydrophobic forces, leading to contraction above the LCST.

Apart from NIPAM, NIPMAM and VCL are also commonly utilized as thermoresponsive monomers in the production of microgels. While VCL shares a comparable LCST with NIPAM, it distinguishes itself by providing reduced toxicity and improved biocompatibility, rendering it an appealing substitute for microgel manufacturing. NIPMAM and *N*-n-propylacrylamide (NNPAM) is typically used to tune the LCST for microgels because it have varies LCST (~45 °C for NIPAMAM and ~23 °C for NNPMAM). Balaceanu et al. [43] did a systematically study about the LCST for the microgels with different composition of VCL, NIPAM and NIPMAM monomers. They produced microgels based on VCL while varying the ratios of NIPAM and NIPMAM in different reaction settings. The outcomes showed that VCL/NIPAM microgels displayed distinct LCSTs, each approximately at 35 °C, corresponding to different NIPAM compositions. Conversely, in the case of VCL/NIPMAM microgels, the LCST exhibited a noteworthy increase, extending from 35 °C to 45 °C as the proportion of NIPMAM in the composition was elevated.

Incorporating charged monomers can confer pH and ionic strength responsiveness to microgels. Among these, acrylic acid (AAc) is one of the most commonly utilized anionic comonomers [55], as shown in Figure 2B, imparting pH and ionic strength responsiveness to microgels [53]. When the PNIPAM-*co*-AAc microgel in pH > pKa of AAc (~4.25), the carboxylic acid groups are deprotonated and have negatively charged. The expansion of microgels occurs due to the Coulombic repulsion among the negatively charged groups contained within them. Such microgels also demonstrate sensitivity to changes in ionic strength. The inclusion of different salts in the surroundings generally results in microgel contraction, influenced by complex interactions involving factors like osmosis, charge screening, and Hofmeister effects.

Moreover, pH responsiveness can be imparted to microgels using alternative anionic monomers, such as Itaconic acid and sodium 4-styrenesulfonate. Similarly, cationic monomers can induce pH responsiveness in microgels, with *N*-(3-aminopropyl) methacrylamide [58] and 4-vinylpyridine [59] serving as common cationic comonomers used in microgel formulations. In acidic conditions, below the pKa, cationic microgels undergo protonation of their cationic groups, resulting in a positive charge and subsequent swelling of the microgel. It is important to highlight that in the synthesis of microgels, both positively and negatively charged monomers can be utilized, enabling the production of microgels with a wide range of charge properties [44]. As an illustrative example, [3-(Methacryloylamino) propyl] dimethyl (3-sulfopropyl) ammonium hydroxide inner salt, commonly known as sulfobetaine, has gained significant attention in recent studies [50,60]. Sulfobetaine molecules incorporate both quaternary ammonium salt positively charged groups and sulfopropyl negatively charged groups. Interestingly, zwitterionic microgels containing sulfobetaine maintain a constant size and do not display pH-dependent swelling behavior due to the permanent presence of both positive and negative charges within their structure [56], as shown in Figure 2C. However, it is important to note that an increase in the amount of zwitterionic monomer leads to higher polydispersity and a decreased volume swelling ratio in these microgels.

Several strategies have been devised to introduce light-responsive properties into microgels, with one approach utilizing the UV-triggered *trans*-to-*cis* behavior [61,62,63]. For instance, 4-[(4-methacryloyloxy) phenylazo] benzenesulfonic acid (ABSA) undergoes rapid transformation from a *trans* state to a *cis* state upon exposure to UV irradiation [57]. When in its *trans* state within the microgel, ABSA demonstrates linear behavior, enabling water molecules to move freely, consequently enhancing microgel hydration and resulting in an increased size. However, upon UV irradiation-induced transformation of ABSA from *trans* to *cis*, ABSA molecules undergo folding, which intensifies hydrophobic interactions and leads to microgel shrinkage, as illustrated in Figure 2D. Another strategy involves the use of monomers like Nitrobenzylmethacrylate (NBMA), which can be cleaved upon UV irradiation [64]. In this investigation, scientists incorporated hydrophobic drug molecules into a photoresponsive microgel. When exposed to UV irradiation, NBMA molecules experienced cleavage, generating carboxylic acid groups within the microgel. This, in turn, enabled the expulsion of all the hydrophobic drugs from the microgel.

Heavy metal ion capture groups can also be incorporated into microgels to confer upon them metal ion-responsive properties. Crown ethers, in particular, are extensively employed for their efficacy in capturing metal ions [65]. Luo et al. designed a highly sensitivity and selectively microgels for Pb^2+^ ions [54]. In this study, the researchers synthesized a microgel containing 18-crown-6 crown ether, which selectively binds to Pb^2+^ ions, as illustrated in Figure 2E. When the crown ether captures a Pb^2+^ ion in the solution, the repulsion of charges leads to the swelling of the microgel.

Instead of the previously mentioned monomer, a variety of functional monomers can be utilized in the synthesis of stimuli-responsive microgels. These functional monomers confer unique responsive properties to the resulting microgels, rendering them suitable for diverse research applications. Although we will not explore all of them in this discussion.

### 2.2. Cross-Linker Chemistry

Microgels are stable colloidal dispersions composed of cross-linked polymeric particles [66]. The gel network is upheld through the presence of cross-linkers, which serve to inhibit the dissolution of polymer chains into the solvent. The additive of cross-linkers between polymer chains affects the physical properties of the polymer such as elasticity [67,68], viscosity [68,69,70], and glass transition temperature [68,71]. As the cross-link concentration increases, the polymer becomes more rigid, its viscosity decreases, and its glass transition temperature rises. These changes can be attributed to the restrictions placed on the mobility of polymer chains. Moreover, the cross-linking density also influences the pore size of microgel particles [72]. Increased cross-linking density results in a reduction in pore size. Cross-linking can be broadly classified into two main categories: chemical and physical cross-linking. Chemical cross-linking involves the creation of covalent bonds between polymer chains, while physical cross-linking relies on non-covalent interactions like hydrogen bonding, hydrophobic interactions, and ion interactions to maintain the polymer network.

#### 2.2.1. Chemical Cross-Linking

Chemical cross-linking can take place during the radical polymerization process. These cross-linkers contain multiple carbon-carbon double bonds that form covalent bonds with vinyl monomers through radical reactions, leading to the formation of a gel network. For instance, *N*,*N*-methylenebisacrylamide (BIS) is frequently employed in polyacrylamide (PAA) and PNIPAM due to its structural resemblance to the monomer [73]. Ethylene glycol dimethacrylate (EGDMA) and ethylene glycol diacrylate (EGDA) are other widely used water-soluble cross-linkers to synthesize microgels [68,74].

**Figure 3:**
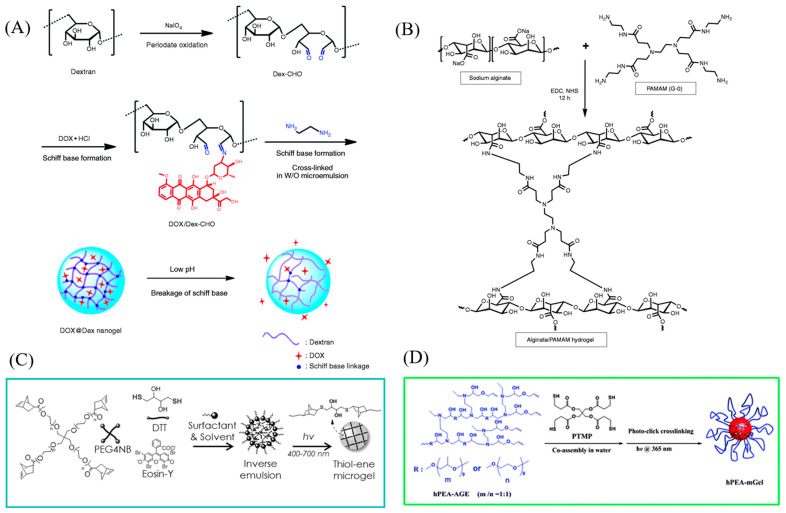
(**A**) Schematic illustration of the synthesis of Schiff base-containing dextran nanogel as carrier system of doxorubicin, drugs would be released efficiently under acidic condition. Reprinted with permission from Ref. [76] (**B**) Synthesis of covalently cross-linked alginate/PAMAM injectable hybrid hydrogel. Reprinted with permission from Ref. [80] (**C**) Schematic of inverse suspension and visible-light-initiated thiol-ene reaction for forming step-growth microgels. Reprinted with permission from Ref. [79] (**D**) Scheme of the process to prepare hPEA-mGel. Reprinted with permission from Ref. [81].

In addition to radical polymerization, cross-linking can be accomplished through chemical reactions. Functional groups found on monomers, such as NH3, COOH, and OH, can form covalent bonds with cross-linker molecules. For example, amine groups can react with aldehyde groups under mild conditions to create Schiff Bases, a mechanism commonly used to cross-link polymer chains. For example, glutaraldehyde is used as a cross-linker to synthesize chitosan microgels [75]. Aldehyded dextran can be cross-linked with ethylenediamine to form dextran-based microgels [76], as shown in Figure 3A.

Condensation reactions that involve carboxylic groups and either amine or hydroxyl groups provide another method for generating cross-links in microgel synthesis. Poly(ethylene glycol)-diamines (PEG-diamines) can act as efficient cross-linkers, establishing covalent bonds with the carboxylic groups on polymer chains through a coupling reaction facilitated by 1-Ethyl-3-(3-dimethylaminopropyl) carbodiimide (EDC). For instance, alginate-based hydrogel/microgel formulations can be prepared by cross-linking with PEG-diamines in this manner [77,78], as shown in Figure 3B.

Thiol groups react with double bonds via thiol-ene addition, which isa widely used method to form cross-links. Dithiothreitol (DTT) is able to cross-link with PEG-tetra-norbornene (PEG4NB) to synthesize PEG-based microgels [79,80], which is shown in Figure 3C. In addition, thiol-ene photo-click cross-linking is also reported between pentaerythritol tetra(3-mercaptopropionate) (PTMP) and allyl group-ended hyperbranched poly(ether amine) (hPEA-AGE) for microgel synthesis (Figure 3D) [81].

#### 2.2.2. Physical Cross-Linking

**Figure 4:**
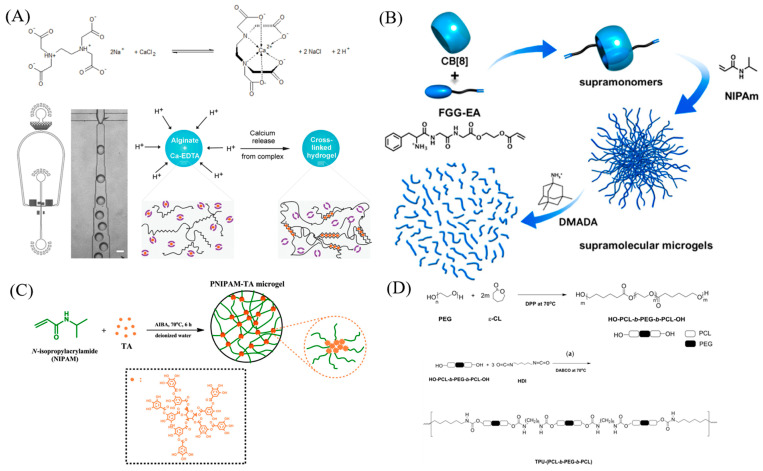
(**A**) Microfluidic generation of homogeneously crosslinked alginate microparticles by on−demand release of calcium ions from a water-soluble Calcium−EDTA complex. Reprinted with permission from Ref. [82] (**B**) Fabrication of Supramolecular Microgels from Supramonomers. Reprinted with permission from Ref. [84] (**C**) Synthetic Routes of PNIPAM−TA Microgels. Reprinted with permission from Ref. [87] (**D**) Synthesis of (top) hydroxyl-group terminated HO-PCL-b-PEG-b-PCL-OH triblock copolymers and (bottom) corresponding TPU-(PCL-b-PEG-b-PCL) thermoplastic polyurethane multi-block copolymers. Reprinted with permission from Ref. [88].

In contrast to chemical cross-linking, physical cross-linking relies on ionic interactions, hydrogen bonding, and hydrophobic interactions rather than covalent bond formation to create a gel network. Notably, ionic interactions are widely employed in the physical cross-linking of microgels. Alginate serves as a prominent example of ionic cross-linking, as shown in Figure 4A. The carboxylate groups present in alginate have the capability to bind with calcium ions, resulting in the formation of a gel network [74,82]. Furthermore, the oxygen atoms within dextran can engage with potassium ions, leading to the formation of cross-links [83].

Supramolecular host-guest interactions provide an alternative approach for forming cross-links in microgel synthesis. As an illustration in Figure 4B, cucurbit[8] uril (CB[8]) can form complexes with acrylate-linked Phe-Gly-Gly (FGG-EA) in a 1:2 ratio, serving as a supramolecular cross-linker [84]. Supramolecular host-guest complexes, like the interactions between β-cyclodextrin and cholesterol, provide an alternative avenue for synthesizing physically cross-linked microgels. By promoting cross-linking between β-cyclodextrin-linked polymer chains and cholesteryl-functionalized PEG, it becomes possible to create degradable microgel particles [85]. Furthermore, tannic acid is capable of forming complexes with VCL monomers [86], PNIPAm monomer [87], through hydrogen bonding providing a means to synthesize physically cross-linked microgels, as shown in Figure 4C.

Amphiphilic block copolymers possess the capability to undergo self-assembly, leading to the formation of micelles within an aqueous solution. This constitutes another category of physically cross-linked microgels [74]. For example, in Figure 4D, a triblock copolymer poly(ethylene glycol-*b*-(DL-lactic acid-*co*-glycolic acid)-*b*-ethylene glycol) (PEG-PLGA-PEG) can form a gel at body temperature [88].

Hydrophobic polymers such as polylactic acid (PLA) and polycaprolactone (PCL) can be blended with the hydrophilic polymer polyethylene glycol (PEG) to produce block copolymers like PEG-PLA and PEG-PCL. These block copolymers are employed in the development of physically cross-linked gel networks [89,90].

### 2.3. Microgels Post-Functionaliztion

Apart from directly incorporating functional monomers and crosslinkers during microgel synthesis, an alternative approach to obtain functional and stimuli-responsive microgels for biomedical applications is through post-functionalization. This process typically involves the conversion of a pre-synthesized functional microgel precursor into its post-functionalized state under specific reaction conditions [91,92]. A variety of chemical reaction tools have been investigated to design and develop post-functionalized, stimuli-responsive microgels, such as carbodiimide crosslinking chemistry [93], click chemistry [94,95], enzyme-mediated conjugation [96], and so on. By means of post-functionalization, various functional moieties, such as small molecules, biomolecules (including peptides, proteins, and nucleic acids), as well as nanomaterials, can be introduced onto the surface and/or within stimuli-responsive microgels [91]. The post-functionalization approach offers several notable advantages in the design and synthesis of innovative and functional microgels. Firstly, it simplifies the process by avoiding the complexities and challenges associated with synthesizing and preparing reactive or polymerizable monomers. Secondly, due to the versatility of post-functionalization techniques, it allows for the modification of diverse functional groups within the same set of microgels, eliminating the need to create distinct functional microgels individually. Lastly, the inherent orthogonality of post-functionalization methods, especially exemplified by click chemistry, enables the introduction of a wide range of moieties into a single microgel. This versatility grants microgels the ability for multiple modes of responsiveness and multifunctionality. Given these advantages, post-functionalization has gained increasing popularity in the realm of designing functional microgels in recent years. In this section, we will delve into and highlight several examples of post-functionalized stimuli-responsive microgels.

#### 2.3.1. Carbodiimide Cross-Linking Chemistry

Carbodiimide cross-linking represents a commonly used coupling reaction that involves the formation of an amide bond through the mediation of a carbodiimide compound between a primary amine group and a carboxylic acid group [97,98,99]. In typical procedures, a carboxylic acid functional group is initially activated using carbodiimide-containing compounds such as carbonyldiimidazole (CDI), *N*,*N′*-dicyclohexyl carbodiimide (DCC), or 1-ethyl-3-(3-dimethylaminopropyl) carbodiimide hydrochloride (EDC). The activation step leads to the creation of an active *O*-acylisourea intermediate. Following this, the intermediate is substituted by a primary amine from the reaction mixture through nucleophilic attack, resulting in the formation of a product containing an amide bond.

**Figure 5:**
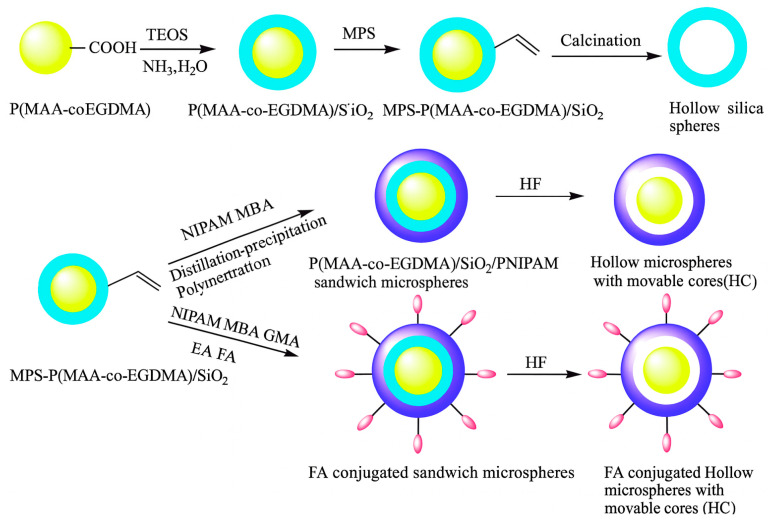
Schematic illustration of the fabrication process of the hollow silica microsphere, the hollow microspheres with movable cores (HC) and FA-conjugated targeting temperature-sensitive hollow microspheres with movable pH-responsive cores. Reprinted with permission from Ref. [100].

Regarding the design of functional microgels, we can propose the following reaction scheme: the microgel precursors may either include carboxylic acid while the functional compound contains a primary amine, or vice versa. This coupling reaction is versatile and straightforward, allowing the preparation of various functional microgels for biomedical applications [91,100,101,102]. Liu and co-workers [100] prepared monodisperse poly(methacrylic acid-co-ethyleneglycol dimethacrylate) (P(MAA-*co*-EGDMA)) microgel microgels exhibited obvious temperature stimulus-responses with diameters related to the degree of crosslinking and monomer proportion, as shown in Figure 5. The research conducted a study to investigate the controlled release of drug molecules, focusing on the anti-cancer drug doxorubicin (DOX). The release was controlled under two independent conditions simulating the human body: temperature and pH. Furthermore, the study assessed the biocompatibility of hollow microspheres sensitive to temperature. These microspheres contained pH-responsive cores and were modified with folic acid (FA) for targeted drug delivery. The results highlight the potential of precise molecular targeting enabled by surface folic acid groups and the dual-stimulus responsiveness of the unique yolk/shell structure. This holds promise for improving cancer treatment strategies.

#### 2.3.2. Click Chemistry

**Figure 6:**
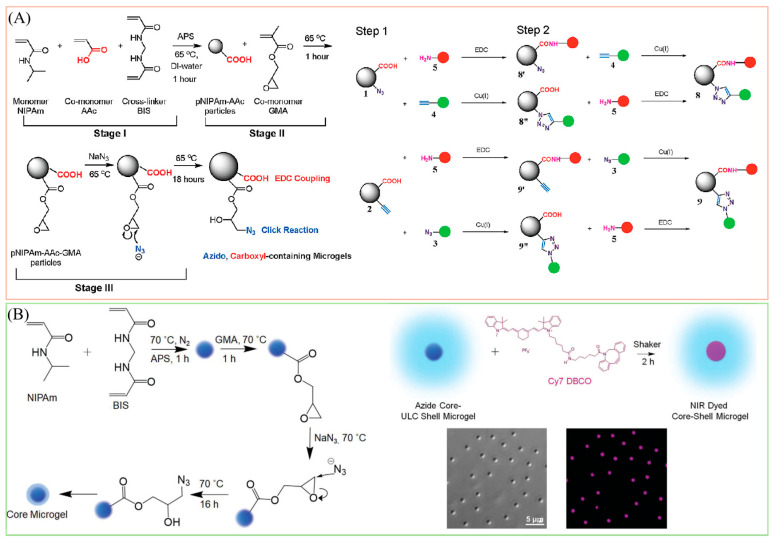
(**A**) One-Pot, Two-Stage Synthesis of Alkynyl- and Carboxylic Acid-Containing Microgels and the two-Step Coupling to Microgels 1 and 2. Reprinted with permission from Ref. [94] (**B**) One-pot, three-step synthesis of azide-containing crosslinked cores. Functionalization of the core–shell microgel with Cy 7 dye by the DBCO-azide reaction and the morphology of the core–shell microgels. Reprinted with permission from Ref. [95].

As the term “click chemistry” was first introduced by Sharpless and co-workers in 2001 [103], it has gained much attention since then. Click chemistry is characterized by a series of chemical reactions known for their distinctive properties, including modularity, broad reaction applicability, stereospecificity, straightforward reaction conditions, and high yield outcomes [104,105,106]. Given these advantageous features and the potential for biocompatibility and biorthogonality, click chemistry has garnered significant attention as a valuable tool for post-functionalizing microgels in diverse applications [107,108]. For instance, the copper-catalyzed azide-alkyne cycloaddition reaction (CuAAC), recognized as one of the most extensively studied click chemistry tools, has been employed by Meng et al. to fabricate multi-responsive microgels (shown in Figure 6A) [94]. In their study, they designed microgels that incorporated clickable functional groups such as azide and alkyne. They demonstrated subsequent orthogonal chemoligation, using alkyne or azide-modified fluorescein as an illustrative example. In a recent instance, Islam et al. developed core–shell microgels with azide groups in the core region, as illustrated in Figure 6B, allowing for the conjugation of various dibenzocyclooctyne-containing fluorophores to the microgels under mild reaction conditions through the strain-promoted azide-alkyne cycloaddition (SPAAC) pathway [95]. Unlike CuAAC, SPAAC presents clear advantages in relation to reaction conditions, notably in its elimination of toxic copper ions as catalysts. Moreover, in addition to the azide-alkyne cycloaddition reactions like CuAAC and SPAAC, researchers are actively exploring and increasingly adopting various other click chemistry methodologies, including thiol-ene reactions, thiol-Michael additions, Diels-Alder reactions, and epoxide ring-opening reactions [91]. It is important to mention that each type of click chemistry tool has its own distinct advantages and disadvantages, and its appropriateness depends on the specific application being studied.

#### 2.3.3. Enzyme-Mediated Conjugation

**Figure 7:**
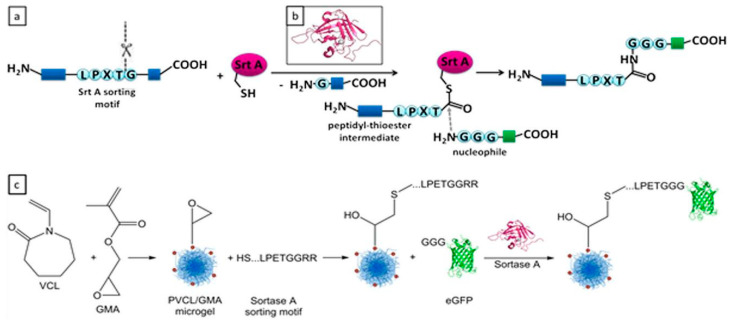
(**a**) Reaction mechanism of sortase-mediated ligation; (**b**) structure of sortase A; and (**c**) microgel synthesis and sortase-mediated postmodification approach. Reprinted with permission from Ref. [96].

The post-functionalization techniques mentioned earlier, classified as chemoligation methods, pose substantial challenges when attempting to attach biomolecules such as proteins and nucleic acids to microgels. These approaches frequently encounter issues related to low efficiency and restricted controllability [109]. In contrast, enzyme-mediated conjugation emerges as an alternative approach that effectively addresses several of the shortcomings associated with chemoligations [110]. Furthermore, enzyme-based methods, naturally occurring in various species, offer the advantage of preserving the native structures and functions of biomolecules [111]. Thanks to the mentioned advantages, enzyme-mediated conjugation methods have witnessed a surge in their utilization for functionalizing various natural or synthetic polymers, including microgels. For example, Gau et al. demonstrated functionalization of the surface of poly(N-vinylcaprolactam) microgels via sortase-mediated method [96]. In their intriguing study, they introduced glycidyl methacrylate into the shells of their microgels, followed by a subsequent reaction with a specific recognition peptide sequence (LPETG) via epoxy-thiol reactions, as depicted in Figure 7. Subsequently, Sortase A enzyme could recognize this specific sequence and facilitate the ligation between the sequence and triglycine-modified Enhanced Green Fluorescent Protein (N3G-eGFP). In summary, this work showcased an enzyme-mediated pathway as an efficient and highly controllable method for post-functionalizing stimuli-responsive microgels.

The design strategy of responsive microgels is a multifaceted approach focused on tailoring the properties and behavior of these microscopic particles to specific applications. Researchers employ a variety of techniques, including the incorporation of responsive monomers, cross-linking methods, and post-functionalization, to impart sensitivity to stimuli such as temperature, pH, light, or ions. These strategies aim to create microgels that can intelligently and reversibly change their size, shape, or properties in response to external cues. The ultimate goal is to engineer microgels with precise and controllable responsiveness, allowing them to serve as versatile platforms for drug delivery, bioimaging, biosensing, and tissue engineering, among other biomedical applications. This design flexibility opens up exciting possibilities for the development of innovative and patient-centric solutions in the field of biotechnology and medicine.

## 3. Conclusion and Perspective

In conclusion, stimuli-responsive microgels have emerged as remarkable smart materials with a profound impact on various biomedical applications. These microscaled hydrogel particles offer an array of advantages, from high water content and biocompatibility to responsiveness to diverse environmental stimuli. Their precise tunability and versatile synthetic methodologies have enabled the development of tailored solutions for drug delivery, bioimaging, biosensing, and tissue engineering [8,9,112]. While substantial progress has been made, the field of stimuli-responsive microgels continues to evolve, presenting exciting opportunities for future exploration. Advanced functionalization, in vivo applications, combination therapies, personalized medicine, and regulatory considerations are all areas that promise significant growth and innovation. As we stand on the threshold of a new era in biomedicine, the potential of stimuli-responsive microgels to revolutionize diagnosis, treatment, and disease understanding is both promising and inspiring. With interdisciplinary collaboration and sustained research efforts, these smart materials will undoubtedly play a pivotal role in shaping the future of healthcare, ultimately benefiting patients worldwide.

In future, the translation of stimuli-responsive microgels from research to clinical practice requires navigating regulatory approval processes. While microgels show great promise in controlled delivery and biomedical engineering, several challenges limit their direct clinical translation. Toxicity arising from residual monomers, crosslinkers, or surfactants, as well as the potential for unintended immunological responses, must be carefully evaluated. Moreover, in vivo clearance pathways-such as renal filtration, hepatic metabolism, or uptake by the mononuclear phagocyte system-can significantly influence therapeutic performance and long-term safety. These factors often differ substantially from in vitro observations, underscoring the need for comprehensive in vivo studies and standardized assessment protocols before clinical application. Collaborations between researchers, clinicians, and regulatory agencies will be essential. Developing scalable and cost-effective methods for producing stimuli-responsive microgels will be necessary to make these therapies accessible to a broader patient population.
